# Genotype by environment interaction for tick resistance of Hereford and Braford beef cattle using reaction norm models

**DOI:** 10.1186/s12711-015-0178-5

**Published:** 2016-01-14

**Authors:** Rodrigo R. Mota, Robert J. Tempelman, Paulo S. Lopes, Ignacio Aguilar, Fabyano F. Silva, Fernando F. Cardoso

**Affiliations:** 1 Department of Animal Science, Universidade Federal de Viçosa, Viçosa, Minas Gerais Brazil; 2 Department of Animal Science, Michigan State University, East Lansing, USA; 3 Instituto Nacional de Investigación Agropecuaria-INIA Las Brujas-Canelones, Rincón del Colorado, Canelones Uruguay; 4 Embrapa South Livestock, Bage, Rio Grande do Sul, Brazil and Department of Animal Science, Universidade Federal de Pelotas, Pelotas, Rio Grande do Sul Brazil

## Abstract

**Background:**

The cattle tick is a parasite that adversely affects livestock performance in tropical areas. Although countries such as Australia and Brazil have developed genetic evaluations for tick resistance, these evaluations have not considered genotype by environment (G*E) interactions. Genetic gains could be adversely affected, since breedstock comparisons are environmentally dependent on the presence of G*E interactions, particularly if residual variability is also heterogeneous across environments. The objective of this study was to infer upon the existence of G*E interactions for tick resistance of cattle based on various models with different assumptions of genetic and residual variability.

**Methods:**

Data were collected by the Delta G Connection Improvement program and included 10,673 records of tick counts on 4363 animals. Twelve models, including three traditional animal models (AM) and nine different hierarchical Bayesian reaction norm models (HBRNM), were investigated. One-step models that jointly estimate environmental covariates and reaction norms and two-step models based on previously estimated environmental covariates were used to infer upon G*E interactions. Model choice was based on the deviance criterion information.

**Results:**

The best-fitting model specified heterogeneous residual variances across 10 subclasses that were bounded by every decile of the contemporary group (CG) estimates of tick count effects. One-step models generally had the highest estimated genetic variances. Heritability estimates were normally higher for HBRNM than for AM. One-step models based on heterogeneous residual variances also usually led to higher heritability estimates. Estimates of repeatability varied along the environmental gradient (ranging from 0.18 to 0.45), which implies that the relative importance of additive and permanent environmental effects for tick resistance is influenced by the environment. Estimated genetic correlations decreased as the tick infestation level increased, with negative correlations between extreme environmental levels, i.e., between more favorable (low infestation) and harsh environments (high infestation).

**Conclusions:**

HBRNM can be used to describe the presence of G*E interactions for tick resistance in Hereford and Braford beef cattle. The preferred model for the genetic evaluation of this population for tick counts in Brazilian climates was a one-step model that considered heteroscedastic residual variance. Reaction norm models are a powerful tool to identify and quantify G*E interactions and represent a promising alternative for genetic evaluation of tick resistance, since they are expected to lead to greater selection efficiency and genetic progress.

## Background

The cattle tick is a parasite that adversely affects beef cattle production in tropical areas such as Brazil. Retail beef markets are imposing restrictions on meat, ensuring that it is free of chemical residues that are perceived as having negative impacts on environment, public health and human welfare. Therefore, to remain competitive in foreign beef markets, Brazil must aim at complying with these higher standards.

To ensure market competitiveness, one strategy might be to increase the contribution of the *Bos taurus* breeds to Brazilian herds because they are more advantageous in terms of productive traits [1], such as carcass yield, gain weight, meat quality and sexual precocity compared to *Bos indicus* breeds. However, *Bos taurus* breeds tend to have greater susceptibility to tick infestation than *Bos indicus* breeds [2, 3]. Hence, selection of animals for tick resistance would be useful to reduce the need for chemical control while also increasing productivity.

Evidence for additive genetic variability of tick counts in cattle includes reported heritability estimates, which range from 0.05 to 0.42 [2, 4–6]. Genetic evaluations for tick counts are routinely performed in countries such as Australia and South Africa, which have a similar climate as Brazil and where the cattle tick is also present. Examples of breeds with such evaluations include breeds such as Bonsmara and Belmont Red [7], and Brahman and Hereford-Shorthorn) [8]. In Brazil, the Conexão Delta G (Delta G Connection) company has used a genetic improvement program based on selection for tick resistance in Hereford and Braford cattle since 2003 [9].

These and other research studies and genetic evaluations [7, 9] have not considered genotype by environment interactions (G*E). Failing to consider G*E interactions in genetic evaluations can adversely affect breeding programs if relative genetic merit is affected by the environment [10–13]; specifically, animals that are identified as top breeders in one environment may not be ideal in other environments. This issue is further exacerbated if progeny are raised in environments that differ from that of their parents [13]. In addition, most current genetic evaluation systems assume homogeneous residual variances across environments, although evidence of residual heteroscedacity has been reported, which is defined as heterogeneity of residual variances across contemporary groups, for traits such as milk yield [14] and post-weaning gain [10, 15].

Linear reaction norm models capture a simple form of G*E interactions. They are based on the use of covariance functions [16] that allow for the prediction of the relative genetic merit of animals as a function of gradual linear changes in an environmental covariate. Sometimes this environmental covariate is not known with certainty and must be estimated from the data; Su et al. [17] demonstrated how this inference uncertainty could be formally accounted for by using Bayesian methods. If G*E interactions are important for tick resistance, reaction norm models could be used to fine-tune genetic improvement for tick resistance in Brazilian beef cattle. Because G*E interactions contribute to heterogeneous genetic variability across environments, if heteroscedastic residual variability across environments is ignored, inferences on G*E interactions based on reaction norm models could be biased.

The objective of this study was to infer upon G*E interactions based on models with different assumptions regarding the nature of genetic and residual variation and with different approaches to account for uncertainty on environmental gradient.

## Methods

### Tick count data

Data used in this current study were obtained from a breeding program conducted by Conexão Delta G (Delta G Connection). Data included records of tick counts (TC) on Hereford and Braford beef cattle from eight herds from the Rio Grande do Sul state, Brazil. TC were obtained on each animal from 326 to 729 days of age using the method described by Wharton and Utech [18], for which all engorged female ticks larger than 4.5 mm were counted on the entire left side of the animal when average management group infestation, i.e., animals under the same feeding and sanitary management, exceeded 20 ticks per animal. Up to three such counts were obtained for each animal, with each count separated by a minimum of 30 days, as described in other studies [5, 19, 20]. A total of 241, 1934 and 2188 animals for which, respectively, one, two and three TC were recorded. The average age during the evaluation period was 524 ± 65 days, and the overall mean TC was equal to 35.0 with a standard deviation of 42.2 (ranging from 0 to 532).

The 4363 animals with records were born between 2008 and 2011 and originated from 604 sires and 3966 dams, with 10 generations of pedigree depth. A total of 11,967 animals remained after pedigree pruning (i.e., removing any terminal ancestors that occur only once in the pedigree file). Pedigree information was incomplete due to the use of multiple-sire matings; 36 % of the animals only had their dam known. For animals with TC, this increased to 65 %. Similar pedigree structures from this same population have been used in other studies [20], and they have not affected the results of genetic evaluation. A detailed breakdown of the pedigree structure is in Table 1.

**Table 1:** Pedigree structure as defined by parentage certainty and pedigree completeness

	With tick counts	Without tick counts	Total
Both parents known	1515	1917	3432
Both parents unknown	4	3807	3811
Only sire known	7	4	11
Only dam known	2837	1876	4713
Total	4363	7604	11,967

**Fig. 1:**
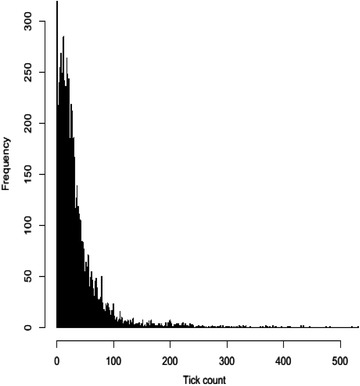
Distribution of tick counts

Because TC were not normally distributed (Fig. 1), a log-transformation was used such that LTTC = log_10_ (TC + 1.001), which was used as the response variable [1, 20]. The constant 1.001 was included because some TC were equal to 0 [1, 20]. Skewness and kurtosis tests were performed and ensured the normality of the residuals from the fitted models.

Contemporary groups (CG) were defined as groups of animals within the same herd, year of birth, season of birth (April–July; August–November and December–March), sex and from the same management group. From 11,316 observations, we selected 10,673 records pertaining to 146 CG with at least five animals and with each LTTC record being within 3.5 standard deviation (SD) from its specific CG. Connectedness among the CG was determined by each having more than 10 genetic links in the dataset, using the AMC software [21]. Estimates of CG effects on LTTC were assumed to be the environmental covariates for a linear reaction norm model because they are the most appropriate entities used to describe the environmental conditions for beef cattle production [10, 22, 23].

### Statistical models

Twelve analyses based on different models and/or inferential methodologies and specifications on residual variability were conducted on the data. These analyses are described below as M_1_ to M_12_ and are summarized in Table 2.

**Table 2:** Statistical model implemented for analysis of tick counts, including approach, contemporary group effect, heteroscedasticity specification, deviance criterion information (DIC) value with respective model ranking

M_x_	Two-step	CG	HET	DIC value	Ranking
M_1_	N/A	R	S_0_	4828.60	12
M_2_	Y	C	S_0_	3736.80	4
M_3_	Y	R	S_0_	4010.93	8
M_4_	N	R	S_0_	3590.84	3
M_5_	N/A	R	S_1_	4507.90	11
M_6_	Y	C	S_1_	3863.92	7
M_7_	Y	R	S_1_	4258.18	10
M_8_	N	R	S_1_	3819.11	5
M_9_	N/A	R	S_2_	4129.39	9
M_10_	Y	C	S_2_	3549.11	2
M_11_	Y	R	S_2_	3823.49	6
M_12_	N	R	S_2_	3114.77	1

*M*_*x*_ model number x; two-step (*Y* yes, *N* no, *N/A* non-applicable because the model is not a reaction norm model); *CG* specification on contemporary group effects (*C* covariate, *R* random classification effect); *HET* heterogeneous residual variance; *S*_*0*_ homoscedastic residual variance; *S*_*1*_ exponential function on heteroscedastic residual variance; *S*_*2*_ discrete subclasses based on classification function on heteroscedastic residual variance

### Traditional animal model (AM)

Consider the following simple linear traditional animal model (M_1_):

1 $$y_{ijk} \,=\,{\mathbf{x}} '_{j} {\varvec{\upbeta}} \,+\, w_{i} \,+ \,a_{j} \, + \, c_{j} \, + \,e_{ijk} .$$

Here, *y*_*ijk*_ is the *k*th LTTC record of animal *j* from CG *i*, **β** is the vector of fixed effects that includes an overall intercept, linear regression coefficients for Nellore breed proportion, heterozygosity and recombination loss (predetermined by Cardoso et al. [9]), as well as linear and quadratic regression coefficients on age of the animal; **x**′_*j*_ is the known incidence row vector of covariates connecting **β** to *y*_*ijk*_; *w*_*i*_ is the random effect of CG *i* ($i = 1, 2, \ldots ,$ 146 levels); *a*_*j*_ is the random additive genetic effect of animal *j*; *c*_*j*_ is the random permanent environmental effect of animal *j*; and *e*_*ijk*_ is the residual error.

The following distributional assumptions were specified:

$${\mathbf{w}} = \, \left\{ {w_{i} } \right\}_{{}} \sim {\text{ N }}({\mathbf{0}},\;{\mathbf{I}}\sigma^{ 2}_{\text{w}} ),$$

$${\mathbf{a}} = \, \left\{ {a_{j} } \right\} \, \sim {\text{ N }}({\mathbf{0}},\;{\mathbf{A}}\sigma^{ 2}_{\text{a}} ),$$

$${\mathbf{c}} = \, \left\{ {c_{j} } \right\} \, \sim {\text{ N }}({\mathbf{0}},\;{\mathbf{I}}\sigma^{ 2}_{\text{c}} )$$

and

$${\mathbf{e}} = \, \left\{ {e_{ijk} } \right\}_{{}} \sim {\text{ N }}({\mathbf{0}},\;{\mathbf{I}}\sigma^{ 2}_{\text{e}} ),$$

where σ_w_^2^, σ_a_^2^, σ_c_^2^ and σ_e_^2^ represent variance components due to CG, additive genetics, permanent environment and residual terms, respectively. Here, **A** represents the numerator of the relationship matrix between the animals in the pedigree, and **I** is the identity matrix.

### Hierarchical bayesian reaction norm models (HBRNM)

Two somewhat different approaches were used to estimate environmental sensitivities of animals. One approach was based on a commonly used two-step model [24, 25], in which in the first step, the regular animal model (M_1_) from Eq. (1) is used to estimate CG effects *ŵ*_*i*_. The second step consists of using these *ŵ*_*i*_ estimates as if they were “known” environmental covariates in a linear reaction norm model. More specifically, posterior means of *ŵ*_*i*_ obtained from M_1_ were used as covariate values in the following reaction norm model (M_2_).

2 $$y_{ijk} \,= \,{\mathbf{x}} '_{j} {\varvec{\upbeta}}\, + \,\phi \hat{w}_{i} \, + \,a_{j} \,+ \,b_{j} \hat{w}_{i} \,+ \,c_{j} \,+ \,d_{j} \hat{w}_{i} \,+ \,e_{ijk} .$$

Here, $\phi$ is an overall linear regression coefficient of *y*_*ijk*_ on *ŵ*_*i*_; *a*_*j*_ is the additive genetic intercept of animal *j* pertaining to genetic merit for an average environment (*ŵ*_*i*_ = 0); *b*_*j*_ is the random additive genetic effect of the reaction norm slope of animal *j* on *ŵ*_*i*_; *c*_*j*_ is the non-genetic (e.g., permanent environmental effect) intercept of animal *j*, as defined for an average environment (*ŵ*_*i*_ = 0); and *d*_*j*_ is the random permanent environmental effect of the reaction norm slope of animal *j* on *ŵ*_*i*_. Note that *y*_*ijk*_, **x**′_*j*_ **β** and *e*_*ijk*_ are defined as before.

Another two-step modeling strategy (M_3_) that is very similar to M_2_ is given by Eq. (3):

3 $$y_{ijk} \,= \,{\mathbf{x}} '_{j} {\varvec{\upbeta}}\, + \,w_{i} \,+ \, a_{j} \,+ b_{j} \hat{w}_{i} \,+ \,c_{j} \,+ \,d_{j} \hat{w}_{i} \,+ \,e_{ijk} .$$

In M_3_, contemporary group effects are refitted as random effects rather than being treated as known covariates, such that M_3_ may be more flexible than M_2_ for modeling CG effects. Nevertheless, *ŵ*_*i*_ was again used as a “known” covariate in the random regression portion of the model.

Including *ŵ*_*i*_ as if it is a “known” covariate in the second step of this approach is clearly a limitation that may understate statistical uncertainty and lead to biased predictions on animal genetic merit. These biases may be due to genetic trend, differences in environmental covariate values across CG, or both [10, 17]. An appealing one-step approach that avoids these limitations of the two-step approach was proposed by Su et al. [17]. This approach is purely Bayesian in that the covariate associated with the reaction norm is treated as unknown, which allows inferences for all unknowns together within a one-step linear reaction norm model (M_4_):

4 $$y_{ijk} \, = \,{\mathbf{x}} '_{j} {\varvec{\upbeta}}\, + \,w_{i} \,+ \,a_{j} \,+ \,b_{j} w_{i} \,+ \,c_{j} \,+ \,d_{j} w_{i} \,+ \,e_{ijk} .$$

Model M_4_ can be rewritten in matrix notation as below [17]:

5 $${\mathbf{y = X\varvec{\upbeta} + Pw + Z}}_{{\mathbf{a}}} {\mathbf{a + Z}}_{{\mathbf{b}}} {\mathbf{b + Z}}_{{\mathbf{c}}} {\mathbf{c + Z}}_{{\mathbf{d}}} {\mathbf{d + e,}}$$

where **y** = {*y*_*ijk*_} is the *nx1* vector of observations; **β** is the vector of fixed effects of order *p*; ${\mathbf{w = }} \, \left\{ {w_{i} } \right\}_{{i{ = }1}}^{{n_{w} }}$ is the vector of environmental effects; **a** = {*a*_*j*_}_*j*=1_^*q*^ is the vector of random genetic intercepts; **b** = {*b*_*j*_}_*j*=1_^*q*^ is the vector of random genetic slopes; **c** = {*c*_*j*_}_*j*=1_^*q*^ is the vector of random permanent environment intercepts; **d** = {*d*_*j*_}_*j*=1_^*q*^ is the vector of random permanent environment slopes; and **e** is the *nx1* vector of residuals. Matrices **X**, **P**, **Z**_**a**_ and **Z**_**c**_ are known incidence matrices, where the column address of matrices **Z**_**b**_ and **Z**_**d**_ has exactly one element equal to the environmental covariate (*w*_*i*_ or an estimate of *w*_*i*_) for that CG in the row address of the observation, with all other elements in that row equal to 0.

### Prior distributional specifications

To infer environmental sensitivities using a hierarchical Bayesian model, three stages are required: the first stage defines the distribution of the observed data conditional on all other parameters [17]:

6 $$\begin{aligned} & {\textbf{y|}}\upbeta , {\textbf{w,a,b,c,d,R}} \sim {\textbf{N}} \hfill \\ & ( {\textbf{X}}\upbeta + {\textbf{Pw}} + {\textbf{Z}}_{\textbf{a}} {\textbf{a}} + {\textbf{Z}}_{\textbf{b}} {\textbf{b}} + {\textbf{Z}}_{\textbf{c}} {\textbf{c}} + {\textbf{Z}}_{\textbf{d}} {\textbf{d,}}\;{\textbf{R)}} .\hfill \\ \end{aligned}$$

For a homoscedastic residual specification such as for M_1_, M_2_, M_3_ and M_4_, **R** = **I**σ_e_^2^, where σ_e_^2^ is the residual variance and **I** is the identity matrix. However, as previously noted, it might be important to model residual heteroscedasticity. We propose two alternative strategies for this. The first heteroscedastic residual specification (S_1_) is defined by **R** = $diag\left( {{\textbf{I}}_{{n_{i} }}\upsigma_{{{\text{e}}_{\text{i}} }}^{ 2} } \right)$, a diagonal matrix with elements equal to $\upsigma_{{{\text{e}}_{\text{i}} }}^{ 2} \,= \,\upsigma_{\text{e}}^{ 2} \times\upeta^{{\hat{w}_{i} }}$ and ${\textbf{I}}_{{n_{i} }}$ denoting an identity matrix of order *n*_*i*_, where *n*_*i*_ is the number of records in the *i*th CG. Here, *η* is an unknown scaling parameter that characterizes the degree of heterogeneity of residual variance across environments, and *ŵ*_*i*_ is the solution for the *i*th CG [26].

Based on S_1_, we tested two two-step approaches (M_6_ and M_7_) that used inferred values of *ŵ*_*i*_ from M_1_ as if they were known and a one-step reaction norm model (M_8_), where *w*_i_ is an unknown covariate that is jointly inferred with the reaction norm and *η* parameters. Model M_6_ was a heteroscedastic residual extension of M_2_, whereas M_7_ and M_8_ were heteroscedastic residual extensions of M_3_ and M_4_, respectively.

Another heteroscedastic residual specification (S_2_) was based on residual variance subclasses determined by a decile-based classification of *ŵ*_*i*_, following Cardoso and Tempelman [10]. That is, CG were ordered into one of 10 categories based on decile delimiters of *ŵ*_*i*_ obtained from M_1_, such that **R** = $diag\left( {{\textbf{I}}_{{n_{k} }}\upsigma_{\text{e}}^{ 2} \upgamma_{k} } \right)$, where the order *n*_*k*_ denotes the number of records delimited by deciles *k* − 1 and *k*, and was 1157, 1174, 1047, 765, 1188, 1192, 1208, 918, 1150 and 874, respectively, for $k = 1, 2, \ldots ,$ and 10. This specification was used to extend the two-step models M_2_–M_10_ and M_3_–M_11_ and the one-step model M_4_–M_12_ with this particular heteroscedastic residual specification.

The last two models considered (M_5_ and M_9_) were heteroscedastic residual animal models based on extending M_1_ with S_1_ and S_2_ heteroscedastic residual specifications, and were used as control models to determine the consequences of failing to model G*E interactions versus failing to model residual heteroscedasticity [10].

The second stage of HBRNM is represented by the prior distributions of the location parameters, as follows:

7 $$\upbeta \sim {\text{p(}}{\varvec{\upbeta}} ),$$

8 $${\mathbf{w}} |\upsigma_{\text{w}}^{ 2} \sim {\text{N(}}{\mathbf{0}} ,{\mathbf{I}}\upsigma_{\text{w}}^{ 2} ),$$

9 $$\left[ {\begin{array}{*{20}c} {\mathbf{a}} \\ {\mathbf{b}} \\ \end{array} } \right]{\sim {\text N}}\left( {\left[ {\begin{array}{*{20}c} {\mathbf{0}} \\ {\mathbf{0}} \\ \end{array} } \right]{\mathbf{,}}\left[ {\begin{array}{*{20}c} {{\varvec{\upsigma}}_{{\mathbf{a}}}^{{\mathbf{2}}} } & {{\varvec{\upsigma}}_{{{\mathbf{ab}}}} } \\ {{\varvec{\upsigma}}_{{{\mathbf{ab}}}} } & {{\varvec{\upsigma}}_{{\mathbf{b}}}^{{\mathbf{2}}} } \\ \end{array} } \right] \otimes {\mathbf{A}}} \right) ,$$

10 $$\left[ {\begin{array}{*{20}c} {\mathbf{c}} \\ {\mathbf{d}} \\ \end{array} } \right]{\sim {\text N}}\left( {\left[ {\begin{array}{*{20}c} {\mathbf{0}} \\ {\mathbf{0}} \\ \end{array} } \right]{\mathbf{,}}\left[ {\begin{array}{*{20}c} {{\varvec{\upsigma}}_{{\mathbf{c}}}^{{\mathbf{2}}} } & {{\varvec{\upsigma}}_{{{\mathbf{cd}}}} } \\ {{\varvec{\upsigma}}_{{{\mathbf{cd}}}} } & {{\varvec{\upsigma}}_{{\mathbf{d}}}^{{\mathbf{2}}} } \\ \end{array} } \right] \otimes {\mathbf{I}}} \right)\text{,}$$

where p(**β**) ∝ 1, σ_w_^2^ is the environmental effect variance; σ_a_^2^ and σ_b_^2^ are the additive genetic variances due to the reaction norm intercept and slope, respectively; σ_c_^2^ and σ_d_^2^ are permanent environment variances due to reaction norm intercept and slope, respectively; σ_ab_ is the genetic covariance between reaction norm intercept and slope; and σ_cd_ is the permanent environment covariance between reaction norm intercept and slope. Then, $\text{r}_{{\text{ab }}} = \upsigma_{\text{ab}} / \sqrt {\upsigma_{\text{a}}^{ 2} \times }\upsigma_{\text{b}}^{ 2}$ and ${\text{r}}_{\text{cd }} = \upsigma_{\text{cd}} / \sqrt {\upsigma_{\text{c}}^{ 2} \times }\upsigma_{\text{d}}^{ 2}$ are the corresponding genetic and permanent environment correlations between intercept and slope, respectively.

Finally, the third stage of HBRNM was based on specifying an inverted gamma (IG) distribution for the variance of the contemporary group effects, i.e., $\upsigma_{\text{w}}^{ 2} | \upalpha_{\text{w}} ,\upbeta_{\text{w}} \sim {\text{IG\,(}}\upalpha_{\text{w}} = 1, \,\upbeta_{\text{w}} = 0 . 0 9 7 )$, where the mean of this distribution is:

11 $${\text{E}}\left( {{\text{w}}_{\text{i}} |\upalpha_{\text{w}} ,\upbeta_{\text{w}} } \right) = \frac{{\upalpha_{\text{w}} }}{{\upbeta_{\text{w}} }} .$$

Similarly, we specify $\upsigma_{\text{e}}^{ 2} | {{\alpha }}_{\text{e}} ,\upbeta_{\text{e}} \sim {\text{IG\,(}}\upalpha_{\text{e}} = 1 , \upbeta_{\text{e}} = 0 . 0 7 2 8 )$.

Likewise, an inverted Wishart distribution (IW) prior distribution was specified for the permanent environment and additive genetic covariance matrices, as follows:

12 $${\mathbf{G}}_{{\mathbf{0}}} \,= \,\left[ {\begin{array}{*{20}c} {{\varvec{\upsigma}}_{{\mathbf{a}}}^{{\mathbf{2}}} } & {{\varvec{\upsigma}}_{{{\mathbf{ab}}}} } \\ {{\varvec{\upsigma}}_{{{\mathbf{ab}}}} } & {{\varvec{\upsigma}}_{{\mathbf{b}}}^{{\mathbf{2}}} } \\ \end{array} } \right] \sim {\text{IW(}}{\mathbf{T}}_{{\mathbf{0}}} , {\text{v),}}$$

13 $${\mathbf{U}}_{{\mathbf{0}}} \,= \,\left[ {\begin{array}{*{20}c} {{\varvec{\upsigma}}_{{\mathbf{c}}}^{{\mathbf{2}}} } & {{\varvec{\upsigma}}_{{{\mathbf{cd}}}} } \\ {{\varvec{\upsigma}}_{{{\mathbf{cd}}}} } & {{\varvec{\upsigma}}_{{\mathbf{d}}}^{{\mathbf{2}}} } \\ \end{array} } \right] \sim {\text{IW(}}{\mathbf{T}}_{{\mathbf{1}}} , {\text{v)}} .$$

Here, *v* = 4 represents a presumed known number of degrees of freedom, and $${\mathbf{T}}_{{\mathbf{0}}} = \left[ {\begin{array}{*{20}c} { 0. 0 1 8 1} & { 0. 0 1 0 2} \\ { 0. 0 1 0 2} & { 0. 0 1 6 1} \\ \end{array} } \right]^{ - 1}$$ and $${\mathbf{T}}_{{\mathbf{1}}} = \left[ {\begin{array}{*{20}c} { 0.0156} & { 0. 0 0 8 7} \\ { 0. 0 0 8 7} & { 0 . 0 1 3 6} \\ \end{array} } \right]^{{ - 1}}$$ are presumed scale matrices for additive genetic and permanent environmental effects, respectively, and $E\left( {{\mathbf{G}}_{{\mathbf{0}}} } \right) = \frac{{{\mathbf{T}}_{{\mathbf{0}}}^{{{\mathbf{ - 1}}}} }}{v - p - 1}$ and $E\left( {{\mathbf{U}}_{{\mathbf{0}}} } \right) = \frac{{{\mathbf{T}}_{{\mathbf{1}}}^{{ - {\mathbf{1}}}} }}{v - p - 1}$ are the prior means for *v* > *p*+*1*, where *p* is the number of parameters In the models with heterogeneous residual variances, additional hierarchical specifications were required, depending on the nature of the function (S_1_ or S_2_) chosen, i.e.: $\upeta | \upalpha_{\upeta} ,\upbeta_{\upeta} \sim {\text{p(}}\upeta |\upalpha_{\upeta} ,\upbeta_{\upeta} ) = {\text{ IG(}}\upalpha_{\upeta} ,\upbeta_{\upeta} )$, for S_1_or $\upgamma_{\text{k}} | \upalpha_{\upgamma} \sim {\text{p}}\left( {\upgamma_{\text{k}} |\upalpha_{\upgamma} } \right) = {\text{IG}}\left( {\upalpha_{\upgamma} ,\upalpha_{\upgamma} - 1} \right)$, $k = 1, 2, \ldots , 10$ for S_2_ [10, 27, 28]. We specified α_η_ = −1 and β_η_ = 0, where the prior p(α_γ_) on α_γ_ was a gamma with shape and scale hyperparameter values of 0.03 and 0.01, respectively [10]. This assumption leads to a prior mean of α_γ_ equal to 3 [E(α_γ_) = 3] and a large prior variance (${\text{var(}}\upalpha_{\gamma } ) = {300}$) [27].

Due to the absence of relevant previous knowledge, flat or highly dispersed prior densities were assumed for all parameters of all models, and hyperparameters for variance components priors were specified on the basis of REML estimates obtained by M_1_ and M_2_ (not shown).

### Bayesian inference

Bayesian analyses were conducted to sample all parameters from their fully conditional posterior distributions. Gibbs sampling was generally used except for the *w*_*i*_’s and *η* in M_5_, M_6_, M_7_ and M_8_ and for α_γ_ (S_2_) in M_9_, M_10_, M_11_ and M_12_. MCMC sampling of these parameters required a random walk Metropolis–Hastings step because their full conditional posterior distributions were unrecognizable (see Cardoso and Tempelman [10] for further details).

Monte Carlo Markov chain (MCMC) based inferences were implemented using the INTERGEN software [29] by saving every 10th cycle from a total of 1,000,000 cycles, after 100,000 cycles of burn-in. Global convergence was checked using Geweke’s Z criterion [30] applied to the conditional distribution of the data, as proposed by Brooks and Roberts [31]. In addition, visual inspection of trace plots was conducted, and a minimum effective sample size of 100 for all unknown parameters was obtained.

### Model comparison

The deviance information criterion (DIC) was used to compare model fit and model complexity [32]:

14 $${\text{DIC}} = \overline{{\text{D}}} \left( {\uptheta } \right)+ {\text{ p}}_{{\text{D}}} = 2\overline{{\text{D}}} {(\uptheta )} - {\text{D(}}\overline{{\uptheta }} ),$$

where $\rm {\overline{D}} (\uptheta ){\text{ }} = {\text{ }}E_{{\uptheta |y}} [D(\uptheta )]$ is the posterior expectation of Bayesian deviance; ${\text{p}}_{\text{D}} \,= \overline{\text{D}} \left(\uptheta \right) - {\text{D(}}\overline{\uptheta} )$ corresponds to the penalty for increasing model complexity, with θ being the vector of model parameters and ${\text{D(}}{\bar{\theta }} )$ being the Bayesian deviance as a function of the posterior mean of the parameters. Smaller values of DIC thereby indicate better-fitting models, while taking a penalty for model complexity into consideration.

### Variance components and genetic parameters

The additive genetic variance of TC for a specific environment *i* with effect *w*_*i*_ was obtained as follows:

15 $$\upsigma _{{\text{a}}}^{2} |{\text{w}}_{{\text{i}}} {\text{ = var}}\left( {{\text{a}}_{{\text{j}}} {\text{ + b}}_{{\text{j}}} {\text{w}}_{{\text{i}}} } \right) = {\mkern 1mu} \upsigma _{{\text{a}}}^{2} {\text{ + w}}_{{\text{i}}}^{2} \,\upsigma _{{\text{b}}}^{2} {\text{ + 2w}}_{{\text{i}}} \;\upsigma _{{{\text{ab}}}} .$$

Thus, the heritability (h_a_^2^) and repeatability (r) of TC for a specific environment was determined as:

16 $${\text{h}}_{\text{a}}^{ 2} | {\text{w}}_{\text{i}} \,= \,\frac{{\upsigma_{\text{a}}^{ 2} | {\text{w}}_{\text{i}} }}{{\upsigma_{\text{a}}^{ 2} \left| {{\text{w}}_{\text{i}} \,+ \,\upsigma_{\text{c}}^{ 2} } \right|{\text{w}}_{\text{i}} \, + \,\upsigma_{\text{e}}^{ 2} | {\text{w}}_{\text{i}} }} ,$$

and

17 $${\text{r|w}}_{\text{i}} = \frac{{\upsigma_{\text{a}}^{ 2} | {\text{w}}_{\text{i}} + \upsigma_{\text{c}}^{ 2} | {\text{w}}_{\text{i}} }}{{\upsigma_{\text{a}}^{ 2} \left| {{\text{w}}_{\text{i}} + \upsigma_{\text{c}}^{ 2} } \right|{\text{w}}_{\text{i}} + \upsigma_{\text{e}}^{ 2} | {\text{w}}_{\text{i}} }} ,$$

where σ_c_^2^|w_i_ and σ_e_^2^|w_i_ are permanent environment and residual variances in environment *i*, respectively. For homoscedastic residual models (from M_1_ to M_4_), σ_e_^2^|w_i_ is constant, i.e., σ_e_^2^|w_i_ = σ_e_^2^∀i. For heteroscedastic residual models, $\upsigma_{{{\text{e}}_{\text{i}} }}^{ 2} | {\text{w}}_{\text{i}} \,= \,\upsigma_{\text{e}}^{ 2} \times\upeta^{{\hat{w}_{i} }}$ for M_5_–M_8_, and $\upsigma_{{{\text{e}}_{\text{i}} }}^{ 2} | {\text{w}}_{\text{i}} \,= \,\upsigma_{\text{e}}^{ 2} \times\upgamma_{k:i}$, where *k*:*i* denotes the decile-based classification *k* for CG *i*, in models M_9_, M_10_, M_11_ and M_12_.

The genetic covariance of TC between two environmental gradients based on covariate values *w*_*i*_ and *w*_*i*’_ was calculated as:

18 $$\begin{aligned} {\text{cov}}_{\text{a}} \left( {{\text{a}}_{\text{j}} {\text{ + b}}_{\text{j}} {\text{w}}_{\text{i}} , {\text{a}}_{\text{j}} {\text{ + b}}_{\text{j}} {\text{w}}_{{{\text{i}}^{ '} }} } \right)\;{ = }\;\upsigma_{\text{a}}^{ 2} { + }\left( {{\text{w}}_{\text{i}} {\text{ + w}}_{{{\text{i}}^{ '} }} } \right)\upsigma_{\text{ab}} {\text{ + w}}_{\text{i}} {\text{w}}_{\text{i}^{\prime}}\upsigma_{\text{b}}^{ 2} ,\hfill \\ \hfill \\ \end{aligned}$$

so that the corresponding correlation between TC in two specific environments was calculated as described below:

19 $$\begin{aligned} {\text{r}}_{\text{a}} \left( {{\text{a}}_{\text{j}} {\text{ + b}}_{\text{j}} {\text{w}}_{\text{i}} , {\text{a}}_{\text{j}} {\text{ + b}}_{\text{j}} {\text{w}}_{\text{i}^{\prime}} } \right)\; = \;\frac{{{\text{cov}}_{\text{a}} \left( {{\text{a}}_{\text{j}} {\text{ + b}}_{\text{j}} {\text{w}}_{\text{i}} , {\text{a}}_{\text{j}} {\text{ + b}}_{\text{j}} {\text{w}}_{\text{i}^{\prime}} } \right)}}{{\sqrt { (\upsigma_{\text{a}}^{ 2} {\text{ + w}}_{\text{i}}^{ 2}\upsigma_{\text{b}}^{ 2} {\text{ + 2w}}_{\text{i}^{\prime}}\upsigma_{\text{ab}} ) (\upsigma_{\text{a}}^{ 2} {\text{ + w}}_{\text{i}^{\prime}}^{ 2}\upsigma_{\text{b}}^{ 2} {\text{ + 2w}}_{\text{i}^{\prime}}\upsigma_{\text{ab}} )} }} .\hfill \\ \hfill \\ \end{aligned}$$

### Estimated breeding values

An estimate of the breeding value of sire *j* for TC, specific to a given environment *i* was obtained by ${\hat{\text{g}}}_{\text{j}} | {\hat{\text{w}}}_{\text{i}} \,= \,\widehat{\text{a}}_{\text{j}} \,+ \,\widehat{\text{b}}_{\text{j}} \widehat{{\text{w}_{i} }}$ [10]. On the one hand, estimates of $\hat{b}_{j}$ close to 0 indicate that *ĝ*_*j*_ is relatively constant across various environments (*ŵ*_*i*_) such that sire *j* has an environmentally robust genetic merit. On the other hand, an environmentally sensitive genetic merit has a large estimate $\hat{b}_{j}$, meaning its relative performance should substantially change on the environmental gradient [33].

The sire breeding value estimates were compared based on the ranking of the animals obtained by AM and HBRNM for low, medium and high environmental levels. These values were defined by the 10, 50 and 90th percentiles for *ŵ*_*i*_. Potential differences in re-rankings of sires for selection based on these models were also determined by the Spearman correlation between estimated breeding values. Spearman correlations were obtained for all animals and for the top 10 % (60) of sires with 12 or more progeny between low, medium and high environmental levels under different fitted models.

## Results and discussion

### Model comparison

Models M_1_, M_5_ and M_9_, which were the only models that did not include G*E interactions with a linear reaction norm model, along with M_7_, and M_1_, had the highest or lowest DIC values. Comparison of DIC between models M_1_, M_5_ and/or M_9_ implies that considering heterogeneity of residual variance across environments is important for modeling LTTC. However, these DIC improvements from homoscedastic to heteroscedastic residual models were small compared to the improvements in DIC when going from regular animal to linear reaction norm models. This suggests that modeling G*E interactions is more important than modeling heterogeneous residual variances (Table 2).

The two one-step reaction norm models (M_4_ and M_12_) had lower DIC values than the corresponding two-step reaction norm models, except for M_10_. Thus, treating all CG effects as uncertain when modeling G*E interactions based on reaction norms seems to be important. This observation is in agreement with the findings of Su et al. [17], who demonstrated by simulation that jointly estimating all unknown parameters is more reliable than using previously estimated environmental effects from a simple animal model as known covariates. DIC can be used to compare any type of model (not necessarily nested models) [10, 13, 22, 23]. However, when fitting two-step models, the reported DIC values come from the second step because we could not account for the uncertainty about *ŵ*_*i*_ estimates from the first step model M_1_. This limitation might have yielded downwards-biased *p*_*D*_ and DIC values for two-step models, but even so, their fit was much poorer compared to their counterpart one-step models (Table 2).

Model M_12_ had the lowest DIC value (Table 2). Recall that M_12_ allows for residual variance groupings into decile-based subclasses, which agrees with the findings of Cardoso and Tempelman [10], who reported this same model as the best-fitting in the characterization of post-weaning gain in Angus cattle.

### Inferences on contemporary group effects

**Fig. 2:**
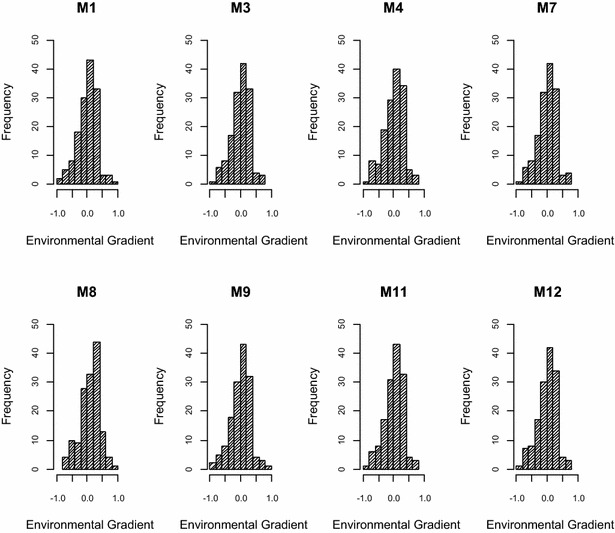
Distribution of the frequencies of environmental gradient estimates (posterior means for contemporary group effects) based on different models

Model M_1_ estimated that CG posterior means (*ŵ*_*i*_) ranged from −0.849 to 0.805, which were considered fixed covariates for models M_2_, M_6_ and M_10_ (Fig. 2). Going from the 0–1st to the 9–10th deciles, corresponding values of *ŵ*_*i*_ were equal to −0.424, −0.224, −0.121, −0.032, 0.032, 0.107, 0.182, 0.240 and 0.316, respectively. Following Cardoso and Tempelman [10], these values were used as the cutoff points for the decile-based heteroscedastic residual subclasses defined in M_9_, M_10_, M_11_ and M_12_.

Posterior means *ŵ*_*i*_ of *w*_*i*_ were similar for all models, regardless of whether G*E interactions were considered, as in M_3_, M_4_, M_7_, M_8_, M_11_ and M_12_, or not, as in M_1_ and M_9_ (Fig. 2); Pearson correlations among these estimates between methods always exceeded 0.99, which means they were also not influenced by homoscedastic versus heteroscedastic residual modeling. These results do not agree with Cardoso and Tempelman [10] for post-weaning gain in Angus cattle, for which estimates *ŵ*_*i*_ from the model with the decile-based heteroscedastic classification function (S_2_) had substantially lower correlations with estimates from the heteroscedastic exponential function models (S_1_), or even the conventional animal models. Furthermore, every model resulted in negative skewness on the *ŵ*_*i*_, ranging from −0.521 to −0.415.

### Inferences on variance components and genetic parameters

The one-step model M_12_ resulted in the highest (0.022 ± 0.04) estimate of the reference or intercept genetic variance (σ_a_^2^) compared with all other models, except for M_8_ (0.025 ± 0.03; Table 3). In addition, M_12_ showed the highest estimate of the genetic variance for slope (σ_b_^2^) compared to the two-step models, except for M_3_ and M_8_, which had the same estimate (0.046 ± 0.022). Estimates of the variance components for reference permanent environment (PE) (σ_c_^2^) were similar among all models (ranging from 0.006 to 0.010). In agreement with σ_b_^2^, PE slopes (σ_d_^2^) were also significant (ranging from 0.015 to 0.084). These results show that the one-step approach confirmed the presence of G*E interactions. Biegelmeyer [20], in a study on tick resistance in Hereford and Braford beef cattle reported similar estimates, i.e., 0.012 and 0.022 for σ_a_^2^ and σ_c_^2^, respectively.

**Table 3:** Posterior means and 95 % posterior probability intervals reported as (2.5, 97.5th) posterior percentiles of dispersion parameters estimated for tick counts of Hereford and Braford cattle by different models

Parameter	Models					
	M_1_	M_2_	M_3_	M_4_	M_5_	M_6_
σ_a_^2^	0.019	0.019	0.020	0.020	0.018	0.019
	(0.011, 0.026)	(0.012, 0.025)	(0.013, 0.026)	(0.013, 0.026)	(0.011, 0.025)	(0.013, 0.025)
σ_b_^2^	N/A	0.032	0.046	0.036	N/A	0.036
		(0.006, 0.074)	(0.011, 0.094)	(0.008, 0.087)		(0.009, 0.069)
σ_c_^2^	0.010	0.008	0.008	0.007	0.010	0.008
	(0.003, 0.018)	(0.003, 0.014)	(0.003, 0.015)	(0.002, 0.014)	(0.003, 0.017)	(0.003, 0.015)
σ_d_^2^	N/A	0.063	0.053	0.084	N/A	0.04
		(0.022, 0.098)	(0.009, 0.093)	(0.031, 0.123)		(0.009, 0.075)
r_ab_	N/A	−0.23	−0.19	−0.14	N/A	−0.28
		(−0.69, 0.42)	(−0.65, 0.43)	(−0.65, 0.55)		(−0.73, 0.40)
r_cd_	N/A	−0.09	−0.07	−0.11	N/A	0.33
		(−0.65, 0.62)	(−0.71, 0.74)	(−0.69, 0.60)		
σ_w_^2^	0.099	N/A	0.097	0.101	N/A	(–0.47, 0.88)
	(0.079, 0.126)		(0.076, 0.123)	(0.080, 0.129)		N/A
σ_e_^2^	0.072	0.063	0.064	0.062	0.070	0.064
	(0.069, 0.074)	(0.060, 0.065)	(0.061, 0.066)	(0.060, 0.065)	(0.067, 0.072)	(0.061, 0.067)

	M_7_	M_8_	M_9_	M_10_	M_11_	M_12_
σ_a_^2^	0.018	0.025	0.021	0.020	0.021	0.022
	(0.012, 0.025)	(0.019, 0.030)	(0.013, 0.028)	(0.014, 0.027)	(0.014, 0.028)	(0.014, 0.028)
σ_b_^2^	0.038	0.046	N/A	0.031	0.035	0.046
	(0.013, 0.065)	(0.021, 0.072)		(0.009, 0.057)	(0.010, 0.063)	(0.009, 0.098)
σ_c_^2^	0.009	0.006	0.010	0.010	0.009	0.009
	(0.004, 0.015)	(0.002, 0.010)	(0.004, 0.017)	(0.004, 0.016)	(0.003, 0.016)	(0.003, 0.016)
σ_d_^2^	0.023	0.015	N/A	0.021	0.020	0.055
	(0.005, 0.051)	(0.003, 0.039)		(0.004, 0.049)	(0.004, 0.050)	(0.006, 0.106)
r_ab_	−0.16	−0.45	N/A	−0.28	−0.17	−0.07
	(−0.08, 0.94)	(−0.68, −0.18)		(−0.67, 0.29)	(−0.61, 0.45)	(−0.57, 0.58)
r_cd_	0.63	0.35	N/A	0.53	0.39	0.30
	(−0.08, 0.94)	(−0.51, 0.87)		(−0.36, 0.93)	(−0.58, 0.91)	(−0.50, 0.89)
σ_w_^2^	0.097	0.113	0.098	N/A	N/A	0.097
	(0.076, 0.123)	(0.088, 0.144)	(0.077, 0.125)			(0.076, 0.124)
σ_e_^2^	0.066	0.071	0.074	0.068	0.069	0.065
	(0.063, 0.068)	(0.068, 0.074)	(0.059, 0.099)	(0.055, 0.089)	(0.056, 0.089)	(0.053, 0.085)

*σ*_*a*_^*2*^ reaction norm intercept genetic variance; *σ*_*b*_^*2*^ reaction norm slope genetic variance; *σ*_*c*_^*2*^ reaction norm intercept permanent environment variance; *σ*_*d*_^*2*^ reaction norm slope permanent environment variance; *r*_*ab*_ genetic correlation between intercept and slope of the reaction norm; *r*_*cd*_ permanent environment correlation between intercept and slope of the reaction norm; *σ*_*w*_^*2*^ contemporary group effect (environmental) variance; *σ*_*e*_^*2*^ residual variance

*M*_*1*_ linear animal model; *M*_*2*_ two-step linear reaction norm model; *M*_*3*_ two-step linear reaction norm model with the random contemporary group (CG) effect being re-estimated; *M*_*4*_ one-step linear reaction norm model with homoscedastic residual variance; *M*_*5*_ linear animal model with exponential function on heteroscedastic residual variance; *M*_*6*_ two-step linear reaction norm model with exponential function on heteroscedastic residual variance; *M*_*7*_ two-step linear reaction norm model with exponential function on heteroscedastic residual variance and with the random CG effect being re-estimated; *M*_*8*_ one-step linear reaction norm model with exponential function on heteroscedastic residual variance; *M*_*9*_ linear animal model with classification function grouped in discrete subclasses on heteroscedastic residual variance; *M*_*1*0_ two-step linear reaction norm model with classification function grouped in discrete subclasses on heteroscedastic residual variance; *M*_*11*_ two-step linear reaction norm model with classification function grouped in discrete subclasses on heteroscedastic residual variance and the random CG effect being re-estimated; *M*_*12*_ one-step linear reaction norm model with classification function grouped in discrete subclasses on heteroscedastic residual variance; *N/A* not applicable

Estimates of the correlation between intercept and slope for the additive genetic and permanent environment effects were characterized by a great deal of uncertainty, as shown by the widths of their respective 95 % posterior probability interval (PPI; Table 3). This large uncertainty differs from those of previous studies [10, 25, 34], which estimated large and positive correlations. These differences may in part be caused by the fact that the correlation estimates depend upon the scale used for *ŵ*_*i*_ or because the biological nature of tick counts is different from that of production traits.

**Fig. 3:**
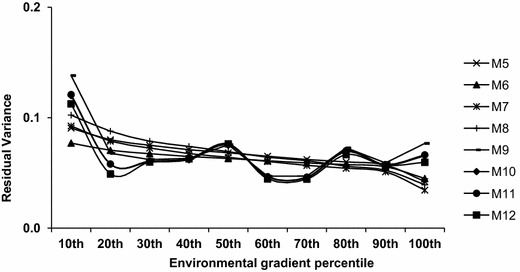
Posterior mean residual variances along the environmental gradient for each 10th percentile of tick counts

Residual variance estimates (σ_e_^2^) were similar among models, ranging from 0.062 ± 0.001 to 0.074 ± 0.010, but they were slightly higher in traditional animal models M_1_ (0.072 ± 0.001), M_5_ (0.070 ± 0.001) and M_9_ (0.074 ± 0.010), which confirms the importance of considering G*E interactions in genetic evaluations for Hereford and Braford beef cattle (Table 3). Cardoso and Tempelman [10] also reported that HBRNM resulted in lower estimates of σ_e_^2^ than AM. However, despite the similarity of the residual variances across the various reaction norm models, Fig. 3 illustrates the need to consider residual heteroscedasticity. The first decile class was particularly deviant from the other classes. This unexpected, very large residual variance at the lowest extreme of the CG effects boundary may be due to data artifacts or a non-obvious biological condition associated with low tick infestation levels. Similar results were demonstrated by Cardoso and Tempelman [10], with residual variances being remarkably decreased at the extremes of the CG average performance. Figure 3 also explains the poor fit of models M_5_, M_6_, M_7_ and M_8_, which modeled heteroscedastic residual variance as an exponential function (i.e., S_1_). This function forced a gradual monotonic change in the residual variances over the CG classes, while M_9_, M_10_, M_11_ and M_12_ showed a more flexible pattern, perhaps reflecting the true residual variance behavior of the actual data.

**Fig. 4:**
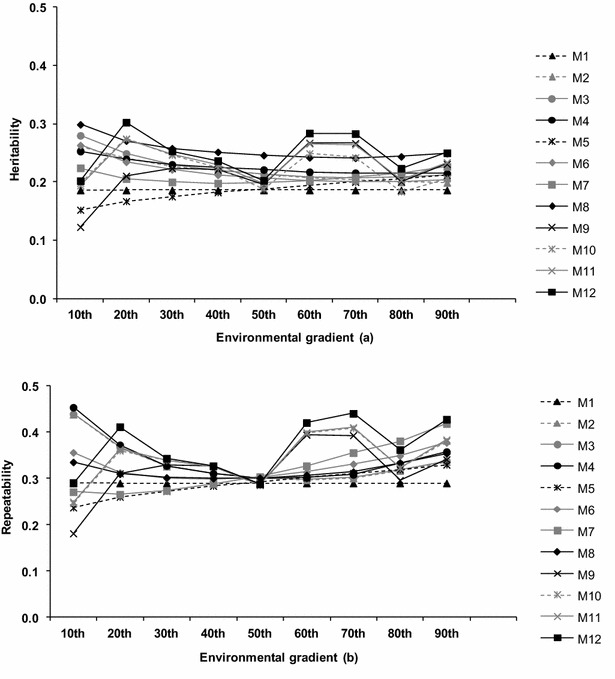
Heritabilities and repeatabilities of tick counts for the 10 and 90th percentiles of the environmental gradient

Heritability estimates ($\hat{h}^{2}$) were generally higher for HBRNM and for M_5_ and M_9_, compared to M_1_ ($\hat{h}^{2}$ = 0.19 ± 0.04; Fig. 4a). Similar heritability estimates have been reported in the literature, using models such as M_1_ and logarithmic transformations of the observed data [1, 5]. With M_12_, average heritability estimates were higher, which also indirectly indicates the better fit of one-step versus two-step models that consider residual heteroscedasticity. Other studies in beef cattle also found higher average heritability estimates for weaning weight and 450-day weight, respectively, using HBRNM compared to AM [10, 35]. Therefore, greater response to selection is expected when using reaction norm models that model heterogeneity of residual variances across CG. Considering that using data from animals with unknown sires could lead to lower heritability estimates, we found that our heritability estimates were similar to those previously reported in the literature [1, 5, 20, 35].

Estimates of repeatability varied along the environmental gradient (ranging from 0.18 to 0.45) and were, in general, higher under high levels of tick infestation (Fig. 4b). These results demonstrate the particular importance of modeling permanent environmental effects in harsh environments, where more resistant animals are more likely to maintain a consistent performance.

**Fig. 5:**
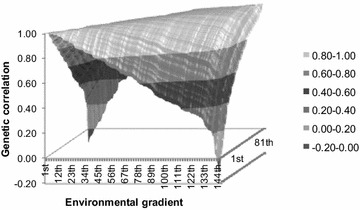
Estimates of genetic correlations between tick counts in different environmental conditions obtained by model M_12_

Posterior means of the genetic correlations [see Eq. (19)] between breeding values along the environmental gradients for Hereford and Braford LTTC that were obtained by the best-fitting model M_12_ demonstrated a large plateau above 0.80 (Fig. 5). Furthermore, estimated genetic correlations decreased as the tick infestation level increased, with negative correlations between extreme environmental levels, i.e., between more favorable (low infestation) and harsh environments (high infestation). Similar results that demonstrate differences in genetic correlations between breeding values along environmental levels, mainly between high challenge conditions and favorable environments, have been reported in the literature [10, 13, 25]. However, Ambrosini [36] estimated small differences for Nellore yearling weight, with genetic correlations between breeding values along the environmental gradient ranging from 0.78 to 1.00.

### Inferences on genetic merit

A low genetic correlation between breeding values in extreme infestation environments (Fig. 5) could indicate that different animals would be selected when using the reaction norm model M_12_. However, Spearman rank correlations among genetic values obtained by different models were always higher than 0.85 (Table 4), which indicates that rankings of animals would be similar and, thus, substantial losses on selection precision might not be observed when using a traditional animal model.

**Table 4:** Spearman rank correlations among posterior means of genetic values for tick counts of Hereford and Braford cattle at different tick infestation levels obtained by different models

Model	M1	M4	M4	M4	M10	M10	M10	M12	M12	M12	M9
Environmental level	(Ov.)	(LTI)	(MTI)	(HTI)	(LTI)	(MTI)	(HTI)	(LTI)	(MTI)	(HTI)	(Ov.l)
M_1_ (Ov.)		0.97	0.98	0.96	0.98	0.99	0.98	0.94	0.97	0.94	0.98
M_4_ (LTI)	0.96		0.97	0.91	0.96	0.97	0.97	0.98	0.96	0.89	0.94
M_4_ (MTI)	0.99	0.97		0.98	1.00	1.00	1.00	0.94	0.99	0.96	0.99
M_4_ (HTI)	0.96	0.91	0.98		0.99	0.98	0.98	0.88	0.97	0.99	0.98
M_10_ (LTI)	0.98	0.96	1.00	0.99		1.00	1.00	0.94	0.99	0.97	0.99
M_10_ (MTI)	0.99	0.97	1.00	0.98	1.00		1.00	0.95	0.99	0.96	0.98
M_10_ (HTI)	0.99	0.97	1.00	0.98	1.00	1.00		0.94	0.99	0.96	0.99
M_12_ (LTI)	0.92	0.98	0.93	0.85	0.92	0.93	0.92		0.95	0.88	0.93
M_12_ (MTI)	0.98	0.97	0.99	0.97	0.99	1.00	0.99	0.94		0.98	0.99
M_12_ (HTI)	0.96	0.91	0.98	1.00	0.98	0.98	0.98	0.86	0.97		0.98
M_9_ (Ov.)	0.99	0.96	0.99	0.98	1.00	0.99	0.99	0.92	0.99	0.98	

Correlations between all animal above the diagonal and between the most used sires (60) below the diagonal

LTI = 10th (−0.424); MTI = 50th (0.032); HTI = 90th (0.316) percentiles of the contemporary group effects (environmental gradient)

*M*_*1*_ linear animal model based on homoscedastic residual variance; *M*_*4*_ one-step linear reaction norm model with homoscedastic residual variance; *M*_*9*_ linear animal model with classification function grouped in discrete subclasses on heteroscedastic residual variance; *M*_*10*_ two-step linear reaction norm model with classification function grouped in discrete subclasses on heteroscedastic residual variance; *M*_*12*_ one-step linear reaction norm model with classification function grouped in discrete subclasses on heteroscedastic residual variance. *LTI* low tick infestation; *MTI* medium tick infestation; *HTI* high tick infestation; *Ov.* overall

## Conclusions

Hierarchical Bayesian reaction norm models can be used to describe the presence of genotype by environment interactions for tick resistance in Hereford and Braford beef cattle. The model that best fitted tick counts in Brazilian climates was a one-step model that considered heteroscedastic residual variance based on ten discrete classes of deciles of average CG performance (M_12_), and hence, this model should be considered as the preferred model for genetic evaluation of this population. However, other functions on residual variance and other classes of models can be evaluated as viable approaches. Reaction norm models are a powerful tool to identify and quantify genotype by environment interactions and present a promising alternative for genetic evaluation of tick resistance, since they are expected to lead to greater selection efficiency and genetic progress.
